# Putting the usability of wearable technology in forensic psychiatry to the test: a randomized crossover trial

**DOI:** 10.3389/fpsyt.2024.1330993

**Published:** 2024-06-14

**Authors:** Peter C. de Looff, Matthijs L. Noordzij, Henk L. I. Nijman, Laurette Goedhard, Stefan Bogaerts, Robert Didden

**Affiliations:** ^1^ Behavioural Science Institute, Radboud University, Nijmegen, Netherlands; ^2^ Science and Treatment Innovation, Fivoor, Rotterdam, Netherlands; ^3^ National Expercentre Intellectual Disabilities and Severe Behavioral Problems, De Borg, Bilthoven, Netherlands; ^4^ Department of Developmental Psychology, Tilburg University, Tilburg, Netherlands; ^5^ Department of Psychology, Health and Technology, Twente University, Enschede, Netherlands; ^6^ Trajectum, Specialized and Forensic Care, Zwolle, Netherlands

**Keywords:** digital mental health, forensic psychiatry, intellectual disability, wearable technology, system usability scale, technology acceptance model, extended confirmation model

## Abstract

**Introduction:**

Forensic psychiatric patients receive treatment to address their violent and aggressive behavior with the aim of facilitating their safe reintegration into society. On average, these treatments are effective, but the magnitude of effect sizes tends to be small, even when considering more recent advancements in digital mental health innovations. Recent research indicates that wearable technology has positive effects on the physical and mental health of the general population, and may thus also be of use in forensic psychiatry, both for patients and staff members. Several applications and use cases of wearable technology hold promise, particularly for patients with mild intellectual disability or borderline intellectual functioning, as these devices are thought to be user-friendly and provide continuous daily feedback.

**Method:**

In the current randomized crossover trial, we addressed several limitations from previous research and compared the (continuous) usability and acceptance of four selected wearable devices. Each device was worn for one week by staff members and patients, amounting to a total of four weeks. Two of the devices were general purpose fitness trackers, while the other two devices used custom made applications designed for bio-cueing and for providing insights into physiological reactivity to daily stressors and events.

**Results:**

Our findings indicated significant differences in usability, acceptance and continuous use between devices. The highest usability scores were obtained for the two fitness trackers (Fitbit and Garmin) compared to the two devices employing custom made applications (Sense-IT and E4 dashboard). The results showed similar outcomes for patients and staff members.

**Discussion:**

None of the devices obtained usability scores that would justify recommendation for future use considering international standards; a finding that raises concerns about the adaptation and uptake of wearable technology in the context of forensic psychiatry. We suggest that improvements in gamification and motivational aspects of wearable technology might be helpful to tackle several challenges related to wearable technology.

## 1 Introduction

Current treatments for forensic psychiatric patients are generally effective, but effect sizes tend to be small to moderate across various relevant outcomes. A meta-analysis (1) on recidivism risk in violent offenders reported that treatments significantly reduced both non-violent and violent recidivism, reporting an odds reduction in (violent) reoffending of approximately 30–35%. Multimodal treatments (intensive cognitive behavioral therapy) were found to be most effective, with significant positive effects on recidivism, although the authors note that the overall effectiveness of psychological treatment on recidivism is small (1). Small effect sizes were also found for the treatment of personality disorders and aggression (2–4), even when e-health innovations were considered (5). Patients in forensic psychiatry often suffer from various (mental) health conditions that severely affect functioning. Examples include, but are not limited to, substance use disorders, depression, bipolar disorder, and schizophrenia (6).

The relatively limited effectiveness of forensic psychiatric treatments can be attributed to the multifaceted nature of violent and aggressive behavior, and complex interactions between psychological, social, environmental, biological and neurophysiological factors (2, 7–9). Special need populations, such as those with mild intellectual disability or borderline intellectual functioning, present additional challenges. Research shows that treatments need to be adjusted to their intellectual and adaptive ability and special needs (10, 11). Personalized and continuous (24/7) treatments tailored to individual needs have been proposed as a promising opportunity to increase the efficacy of current interventions, and improve treatment outcomes (5, 12, 13). Wearable devices show particular promise in transitioning from relatively brief and standardized treatments to continuous and personalized care (14).

Wearable biosensors, such as wristbands, headbands, chest straps and patches (15) provide insight and feedback on physiological signals (e.g., heart rate [variability], breathing rate, temperature, movement, skin conductance). These devices are increasingly being used in the general population and in (mental) healthcare settings to monitor and improve mental and physical health (16). The physiological signals serve as the foundation for creating composite scores or digital biomarkers (17) such as sleep, physical activity or stress indices, which are recognized as transdiagnostic markers of (mental) health and disorders (18–20). The digital biomarkers are typically being created with machine learning methods and artificial intelligence (21). Based on these biomarkers, recommender systems might provide recommendations for personalized interventions, which will have significant impact on the use of the technology in healthcare and the relationship between patients and their healthcare professional (21–23). Recent meta-analyses have resulted in small to medium effect sizes of wearable technology, including fitness trackers, activity trackers and biofeedback devices on stress, sleep, physical activity, depression, emotional and behavioral self-regulation, cardiovascular functioning, and metabolic syndrome (14, 16, 20, 24–31). However, the implementation of wearable technology in forensic psychiatry faces challenges, including limited technology readiness, acceptance, usability of the devices, continuous use of the devices, privacy concerns and data management (14, 32–35).

Three aspects related to the implementation of wearables (usability, acceptance, and continuous use) are deemed crucial for the adoption of the technology (33). Usability serves as a proxy for the ease of use of the devices, and higher scores have been associated with increased adoption and recommendation of new technology (36). Similar findings have been reported for the subjective acceptance of new technology, which consists of two main determinants that relate to the perceived usefulness and ease of use. Finally, continuous use is a proxy for user satisfaction and extended use intention following the purchase or adoption of a product (37).

Multiple use cases for both patients and staff show potential in forensic psychiatric settings. Wearables can be used for monitoring physiological signals, predicting the risk of aggressive and violent behavior (38–40), and distinguishing between different types of violent behavior (32, 41). Biosensors (integrated in wearables) can provide insight into daily-life (physiological) stress reactivity of patients and staff members in different situations (42), and can increase resilience through biofeedback or just-in-time interventions (43). Wearables might also contribute to the overall physical and mental health of patients and staff (25, 26, 30), particularly in forensic psychiatric patients with mild intellectual disability or borderline intellectual functioning (33). That is, if the device and accompanying app is tailored to their needs. However, there is currently a scarcity of research on the usability and acceptance of wearables among special needs samples, such as in individuals with mild intellectual disability or borderline intellectual functioning (44).

Since the introduction of the DSM-5 (Diagnostic and Statistical Manual of Mental Disorders) (45), more emphasis is placed on adaptive functioning (instead of intellectual functioning of IQ) when classifying and assessing the severity of intellectual disability. For instance, borderline intellectual functioning is a V-code, which is used if there is a reason for support or if treatment prognosis is affected (46). The main benefit of wearables for these special needs groups is that the information from the devices might be easily adjusted to their specific needs and capabilities. If implemented correctly, the cognitive load on the user is limited. In addition, individuals might benefit from the non-intrusive and passive monitoring combined with targeted interventions to stimulate physical activity, take rest, or push just-in-time notifications to make them aware of deteriorating sleep-wake patterns and increased stress levels, thereby increasing overall interoceptive awareness and self-regulation (22, 47). For staff members who often deal with challenging and aggressive behavior from the patients, wearables might be beneficial to monitor stress levels, recovery during sleep, and overall health, which might serve as indicators of exhaustion and burnout, but also provide targeted interventions to increase resilience (43, 48). The (continuous) use and acceptance of wearables, both in consumer markets and beyond, have fallen short of initial expectations, especially regarding their continuous adoption on the long term (37, 49, 50). Therefore, prospective studies are imperative to study the usability, acceptance and continuous usage, while considering various use cases, devices, and user preferences. In a previous feasibility study (33), we compared several devices and use cases in forensic psychiatry for patients with mild intellectual disability or borderline intellectual functioning, along with their caregivers. We found an association between actually wearing the device and the intention to continuously use the device. Expectations that people have prior to wearing the device played a relatively minor role in their intention to continuously use the device. One strategy to thus increase adoption and explore various use cases is to let people actually test multiple devices. The previous feasibility study included several proprietary and commercially available devices. An important dilemma that emerged is whether it is worth the effort to develop hardware and software applications for these target groups, or that we might use commercially available devices that are readily available and are optimized for technology readiness and usability, but also have several privacy, judiciary, and proprietary caveats. The feasibility study had limitations: participants wore one device each, which prevented a direct comparison between the devices. Additionally, some devices were worn more often due to the nature of the randomization resulting in an unequal number of participants wearing a particular device.

To address the need for prospective studies and overcome the limitations of the earlier feasibility study (33), we conducted a longitudinal randomized crossover study in which four devices with different functionalities were tested on usability, acceptance, and continuous use. Most previous studies only compared these aspects in separate samples where none of the participants have worn all devices (33, 49, 51). Given the potential applications in forensic psychiatry, we included two general purpose fitness trackers, one device providing real-time bio-cuing in daily life and a device that provides the raw data on multiple physiological signals. The latter is used to provide insight into moment-to-moment and day-to-day physiological reactivity to daily life (stressful) events and situations. The main goal of the current study was to evaluate the usability, acceptance, and continuous use among both patients and staff in forensic psychiatry. We hypothesize that there will be differences in usability, acceptance, and continuous use between devices and user groups (staff vs patients).

## 2 Method

### 2.1 Participants and setting

This study included participants from four Dutch medium security forensic psychiatric centers (i.e., De Borg) that collaborate and are specialized in the treatment of patients with mild intellectual disability or borderline intellectual functioning. Besides their special needs, the patients also suffer from various mental health problems or mental disorders, such as substance use disorder and personality disorder. Many of them display severe aggressive and violent behavior. A total of 32 participants were included, evenly split between patients and staff members. Separate inclusion criteria were determined for patients and staff members. Patients needed to be admitted to one of the forensic wards, had to provide written informed consent, and had to meet eligibility for inclusion as assessed by the head of the multidisciplinary treatment staff. Staff members had to work within one of the forensic centers and have daily interaction with patients. Specific exclusion criteria were acute psychotic state and/or an objection to participation, as assessed by the primary practitioner.

### 2.2 Procedure

The research proposal was approved by the ethics (ECSW2020033) and science committee of Radboud University and adhered to the Helsinki Declaration for research involving human participants. The research was conducted between May 2020 and October 2021. The recruitment process consisted of folders and flyers that were distributed by research coordinators within the four healthcare centers. Participants were informed about the research through these flyers and information leaflets. Upon providing informed consent, participants were enrolled in the study. Each participant wore a different wearable for one week, resulting in a total of four weeks across all four wearables (see 2.3). The order of wearing the devices was randomly determined using a research randomizer to eliminate any ordering effects. The wearables were handed out by research coordinators who provided usage instructions. Prior to wearing the device, participants filled out the usability questionnaire to assess the expected usability of the device. Following the one week wearing period, participants filled out questionnaires to assess usability, acceptance, and continuous usage of the devices. In cases where participants lacked access to a mobile phone or chose not to use it, a research device was provided. If needed, anonymized accounts were created for the participants.

### 2.3 Devices

Four devices were selected in collaboration with staff members who worked on the wards. These choices were guided by considerations such as perceived ease of use, potential benefits for patients and staff members and the applicability to various use cases in forensic psychiatry, such as bio-cueing, emotion regulation, anger management, providing insights, health tracking, or behavior modification. We selected the Fitbit Charge 3, Garmin Vivosmart 4, Empatica E4 (with a custom made user interface currently in active development), and Ticwatch E3 (with a bio-cueing app that is in active development). We opted for the Empatica E4 and Ticwatch E3 with Sense-IT app due to their capacity to provide raw data and ensure anonymous storage of user information, both crucial factors in healthcare, especially in forensic psychiatry (34). While the custom made prototypes for the Empatica and Ticwatch (42, 52, 53) might have lower ease of use and technology readiness scores (54), their inclusion stemmed from being specifically designed for the mental health context. Furthermore, the current study serves as a reference against general purpose fitness trackers.

#### 2.3.1 Fitbit Charge 3

The Fitbit Charge 3 is a fitness tracker equipped with a built-in heart rate monitor that continuously tracks users’ activity levels and heart rate in real-time. Additionally, the accompanying app offers information through composite scores (i.e., digital biomarkers) derived from heart rate and movement sensors, including sleep data. Users receive daily insights on various metrics, including step count, calories burned, stairs climbed and activity metrics. The validity of Fitbit trackers in comparison with golden standard (or criterion) devices varies depending on the physiological signal being tracked and the criterion device used for comparison. For instance, a study showed that the heart rate monitoring of the Fitbit Charge 3 (on photoplethysmography; PPG) in comparison with a criterion chest strap device is relatively poor (see (55)), as indicated by the limits of agreement and poor correlation. Regarding the physical activity measurements, the Fitbit Charge 3 was found to overestimate step count in comparison with a criterion device, though correlations for mean daily step count fall in the moderate to excellent range (56).

#### 2.3.2 Garmin Vivosmart 4

The Garmin Vivosmart 4 is a consumer grade fitness tracker equipped with heart rate monitor (based on PPG) that provides users with various indices of heart rate and accelerometry. In addition, composite scores for sleep, energy expenditure, step count, stairs climbed, and ‘stress’ are available. A systematic review on Garmin activity trackers showed that step accuracy was considered to be good to excellent. However there is a limited number of studies that have assessed the accuracy of sleep, speed or elevation, and these studies often lack a criterion device such as polysomnography for sleep estimation (57). A study conducted with the Garmin Vivosmart 4 in older adults indicated that the device tended to underestimate the step count at low speeds, but exhibited more accurate readings at higher speeds (58).

#### 2.3.3 Empatica E4 with E4 dashboard

The Empatica E4 (59) is a research grade device that records sensor data in a text file (csv) format. It measures blood volume pulse from which heart rate is derived. The E4 also provides an inter-beat-interval to calculate heart rate variability (HRV). HRV indices with the E4 were extensively studied (60–63) and only validated under resting and (very) low movement conditions. Besides blood volume pulse, electrodermal activity (EDA) is recorded and serves as an index for sympathetic nervous system activation (47, 64). EDA is useful for strong and sustained stressors (63), but the reliability and validity compared to criterion devices is uncertain (61). In addition, skin temperature and accelerometer data are recorded. The current study also aimed to further develop an application called the E4 dashboard (42), designed for clinicians and patients to obtain insights into their daily and momentary physiological reactions to stressors and daily events. The dashboard is a precursor to the version described in (42), and presents physiological graphs similar to the graphs provided by Empatica. Participants can add a calendar to the graphs, visualizing physiological reactions during various activities like therapy, conversations, treatment, work or sports. The dashboard also provides clinicians with information on commonly used parameters (e.g., HR, accelerometry, EDA level) and signal quality.

#### 2.3.4 Ticwatch E3 with Sense-IT app

The Mobvoi Ticwatch E3 is a Smartwatch with PPG sensor to measure heart rate, along with an accelerometer and oximeter. In the current study, the full functionality of the Ticwatch was not utilized, but a custom made bio-cueing app called the Sense-IT (52, 53) was installed on the Ticwatch E3. The purpose of the Sense-IT app is to provide real-time and continuous biofeedback on heart rate changes during real-life situations (14). Additionally, it aims to enhance interoceptive and emotional awareness throughout daily activities. It is important to note that usability and validity studies are still in progress with the Sense-IT as it is currently under active development. However, a recent study that used the Sense-IT app indicated that patients and caregivers had a positive attitude towards the application (14), and usability for the Sense-it app ranged from approximately 63 to 76 (65–67). One study indicated that the usability was OK for patients, but staff members generally perceived it as poor (33).

### 2.4 Questionnaires

In collaboration with several staff members who regularly worked with patients, we created revised acceptance and continuous use questionnaires for patients. This adjustment was made to accommodate patients with mild intellectual disability or borderline intellectual functioning who often struggle with word comprehension. To simplify the questionnaire for patients we modified some questions. For example, the question “I would like to use this product frequently” was adjusted to “… more often”. In addition, one question of the System Usability Scale (SUS) was rephrased from a positive statement in the original version to a negatively worded question in the adjusted version (question 3) as this was considered easier for patients to understand.

#### 2.4.1 System Usability Scale

The System Usability Scale (SUS) is commonly used to rapidly assess the subjective usability of a product, including various types of technology such as fitness trackers, digital health applications, or medical devices (36, 68). Usability is the degree to which a product is fit or able to be used. Administration of the questionnaire is considered fast and easy for a plethora of users (69, 70). The SUS has high reliability (*α=.85)* and a meta-analysis (68) showed that usability is a quality feature depending on the ease of use of the applications (and the accompanying technology). Usability scores for physical activity apps were relatively high in the meta-analysis (68), which is of particular interest as this type of application is also used in the current study. The SUS has clear standards and benchmarks (49, 68) in which a total score of ~77 (SD = 15.12) was found across all tested digital health applications in the included studies. The 10-item SUS is scored on a five-point Likert scale ranging from strongly agree (5) to strongly disagree (1). Even numbered questions (items 2, 4, 6, 8, and 10) are negatively worded, while the uneven numbered questions (1, 3, 5, 7, and 9) are positively worded. Missing values are replaced with a 3, following recommendations (71). For positively worded questions, the score minus 1 is calculated, while for negatively worded questions, the score is subtracted from 5. All items are then summed and multiplied by 2.5, effectively yielding a maximum total SUS score of 100. As for the interpretation, a significant body of research is available (36, 69, 72, 73) indicating an average SUS score of 68 being the average among a considerable number of usability scores. People will typically recommend a system that reaches a SUS-score of 82 (73). An adjective scale developed by Bangor et al. (2009) indicated that a mean SUS-score above 35.7 is categorized as having poor usability, above 50.9 is considered to be OK, while a score above 71.4 indicates good usability. Mean scores above 85.5 are considered to be excellent usability scores. We compared the scores in the current study with these benchmarks.

#### 2.4.2 Technology Acceptance Model

The Technology Acceptance Model (TAM) – questionnaire is an often used questionnaire to assess the acceptance of new technology (37). The TAM is based upon the theory of reasoned action (33, 74, 75). For the current study, we administered a more recent development of the TAM that is specifically tailored to smartwatches (37). This TAM version distinguishes between 10 determinants of acceptance summarized in subscales. Two determinants of acceptance are central in the model (75, 76): perceived usefulness (PU) and perceived ease of use (PEOU). These two determinants are central to users’ intention for future technology use and are influenced by the 8 other determinants: mobility, perceptions of and attitudes toward technology, affective quality, subcultural appeal, relative advantage, availability, intention to use, and cost. The TAM has scoring options ranging from strongly agree to strongly disagree on a 7-point Likert scale. The TAM subscales have reliabilities above .70 and the questionnaire consists of 36 questions. For staff members, the full scale was used, whereas a short version with simplified wording was devised for patients. The full questionnaire was perceived to be burdensome to some of them. For each construct, we selected one or two questions of each determinant in cooperation with a team of staff members who frequently interact with these patients. The short TAM questionnaire had a reliability above .80 (Cronbach alpha) (33).

#### 2.4.3 Extended Expectation Confirmation Model

The Extended Expectation Confirmation Model (EECM) is a questionnaire specifically designed to assess the intention for continuous use of smart-wearables (77). The EECM is rooted in the expectation-confirmation theory, which seeks to elucidate user satisfaction in the context of extended use following the purchase or adoption of a product. This model is based on the beliefs that users have with regard to the products’ performance and the (dis)confirmation of these beliefs and expectations (77, 78). The EECM consists of 32 questions that can be scored from strongly agree to strongly disagree using a 7-point Likert scale. The EECM subscales have reliabilities above .70. The 10 subscales of the EECM encompass continuous use, hedonic motivation, battery-life concern, self-socio motivation, perceived privacy, perceived comfort, perceived usefulness, perceived accuracy with functional limitations, satisfaction, confirmation and continuous use. In line with the TAM, we developed a shortened version of the EECM for the patients in the current study. The short EECM has a reliability above .80 (Cronbach alpha) (33). One of the EECM questions was deemed quite difficult for patients to understand due to negation and complex phrasing leading us to rephrase the question into an affirmative one: “I think that the information provided by the product is correct”.

### 2.5 Power analysis

We conducted a power analysis prior to the study, considering an estimated effect size of ~.35 derived from a previous study (33). This previous study investigated the difference between devices for both patients and staff members. To address the research question for a repeated measures-analysis of variance (RM-ANOVA; groups, a power of 95%, and a conservative .25 correlation between measurements, a sample of *n=*28 was needed for the current study. In order to account for drop out, 32 participants were included.

### 2.6 Statistical analysis

First, we calculated descriptive statistics separately for staff members and patients. Subsequently, SUS scores were computed for each device, as well for both groups of patients and staff members. To determine whether there was a difference in usability between devices, a repeated measures ANOVA was conducted with total SUS score as the dependent variable and the type of device as the within-subject factor. Additional models for between-group differences (staff and patients) and interactions were also tested. The TAM and ECCM questionnaires varied in length for patients and staff members, and were therefore analyzed separately using descriptive statistics.

## 3 Results

### 3.1 Sample description

We included 16 patients and 16 staff members who wore all four of the devices and assessed the system usability, technology acceptance and continuous use intention. The age in years of the participants ranged from 18 to 53 (see **Table 1** for an overview of descriptives). Due to technical reasons, the answers of one staff member were not properly saved digitally. To address this, we assigned a value of 3 for the SUS and 4 for both the TAM and the EECM for this participant, in line with recommendations (71).

**Table 1:** Descriptive statistics of sample.

Participants	Patients (n=16), n(%)	Staff (n=16), n(%)
*Education*		
Primary	10(62%)	–
Secondary	6(38%)	5(31%)
Higher	–	11(69%)
*Gender*		
Male	13(81%)	8(50%)
Female	3(19%)	8(50%)
*Age*		
Mean	31.2	33.7
SD	11.5	9.82

### 3.2 Usability (SUS)

After wearing the device, the descriptives from the SUS scores (**Table 2**) indicate that only for staff members, the Fitbit and Garmin devices received a “good” usability rating (36, 72, 73). Regarding patients, the Empatica device with E4 dashboard application was assessed to have poor usability, while the Ticwatch device with Sense-IT application received an “OK” rating. The SUS scores for the Fitbit and Garmin devices increased from the pre-test (expected usability) to the post-test (experienced usability), while the opposite trend was observed for the Empatica and Ticwatch.

**Table 2:** Descriptive statistics of SUS scores.

participant	Product_Start	n	Start	sd	End	sdEnd	minStart	maxStart	minEnd	maxEnd
patient	empatica	16	50.47	15.09	46.09	20.82	30.00	80.00	15.00	75.00
patient	fitbit	16	68.28	14.82	71.25	19.30	42.50	97.50	40.00	100.00
patient	garmin	16	64.69	12.11	70.78	18.77	47.50	87.50	37.50	97.50
patient	ticwatch	16	60.00	13.04	52.66	22.70	40.00	90.00	15.00	87.50
staff	empatica	16	57.66	11.53	55.31	18.93	37.50	77.50	25.00	82.50
staff	fitbit	16	76.25	13.66	76.72	9.52	50.00	100.00	50.00	87.50
staff	garmin	16	64.69	18.77	72.97	13.11	30.00	92.50	47.50	87.50
staff	ticwatch	16	66.09	13.07	56.41	23.13	50.00	92.50	7.50	92.50

To determine differences in system usability between devices for patients and staff, we conducted an RM-ANOVA with the total SUS score as the dependent variable and the type of device as the within subject factor. One outlier was detected for the Fitbit, however, this was not an extreme case. The Shapiro-Wilk test indicated that the data were distributed normally (p>.05). However, sphericity was found to be violated based on Mauchley’s test, χ2(5) = 14.7, *p* = .012. Therefore, we used Huynh-Feldt correction to interpret the results, which returned a significant result, indicating a difference in system usability between devices, *F*(2.631, 81.563) = 18.689, *p* <.001, partial η^2^ = .38. *Post-hoc* analyses with Bonferroni corrections revealed that the Empatica and Ticwatch devices resulted in significantly lower system usability scores in comparison with the Fitbit and Garmin devices. Conversely, there was no significant difference between the Fitbit and Garmin devices on the one hand and Empatica and the Ticwatch on the other.

We also checked (2-way-mixed-anova) differences between staff members and patients on their system usability scores. Unfortunately, the assumptions required for these tests were violated and no non-parametric alternatives were available (79). We were thus unable to formally test the differences between staff and patients. Additional comparisons were non-applicable for age (as it was centered around +/- 30 years), education (we found different distributions for patients and staff members), and gender (there were not many females in the patient group).

### 3.3 Staff acceptance and continuous use

**Figure 1:**
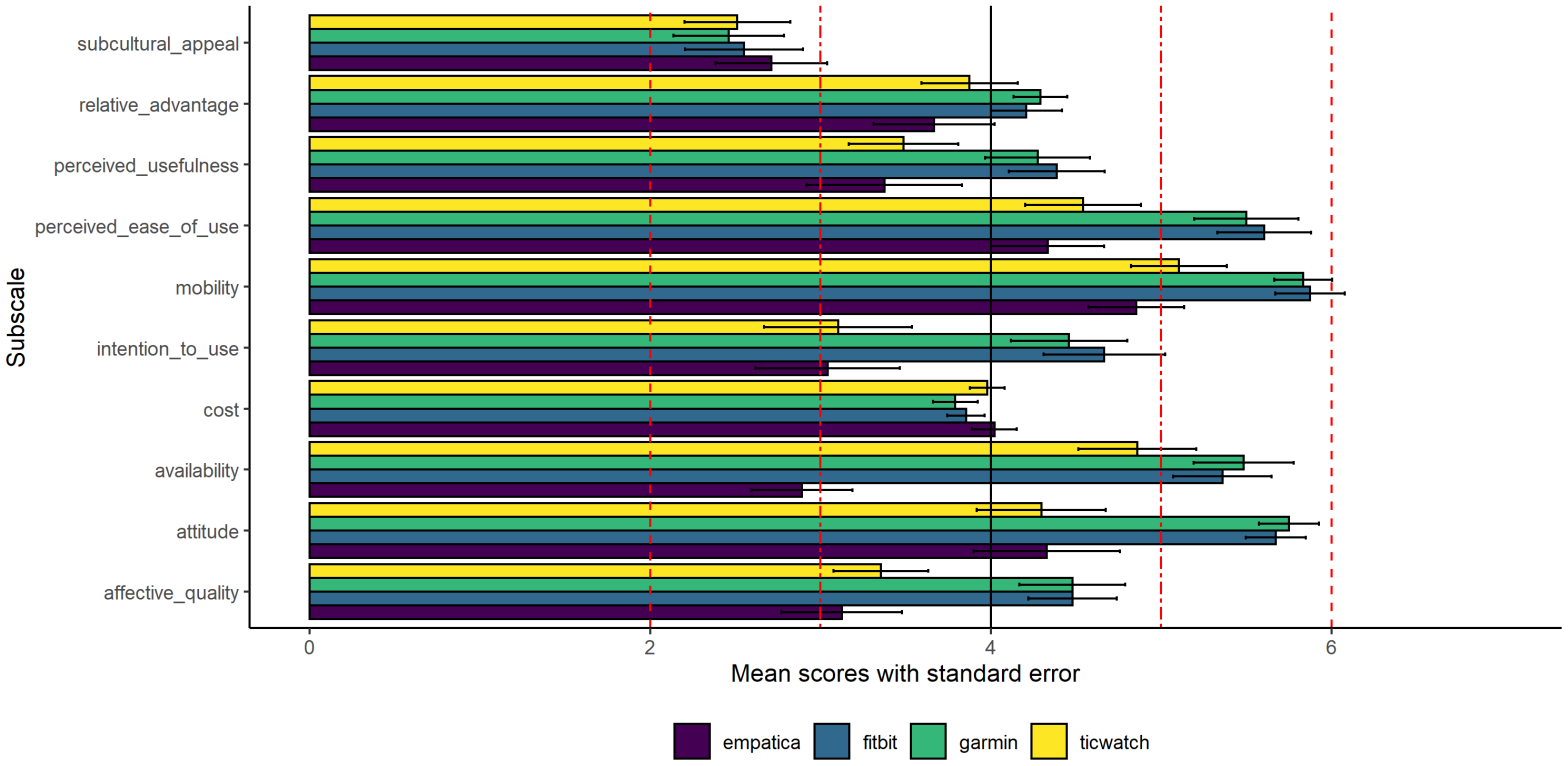
Staff members’ mean scores and standard errors on the subscales of the TAM.

The TAM questionnaire is typically presented in mean or median values on the individual subscales, which differs somewhat from the approach used with the SUS. We have calculated the mean of the subscales for staff members (**Figure 1**), for which perceived ease of use and perceived usefulness are the central subscales. For patients we used a different approach. Since the staff members completed the full version of the TAM, and the patients a shorter version, we cannot directly compare the outcome head to head. Therefore, patients’ results are described in 3.4.

As can be seen in **Figure 1** for the staff members, the subcultural appeal of all devices falls considerably below the median score of the questionnaire. A mean score <3 is indicative of a score below the median, and a mean score >5 is considered well above the median. For the Fitbit and Garmin devices, the perceived ease of use, mobility, availability, and attitude all received ratings are well above the median. However, for the Ticwatch, only the mobility subscale scores are well above the median. The availability of information for the Empatica is considered well below the median, which is probably due to the fact that the wristband has no interface and was not used to provide real-time information during the current study. Information was only available after the participant had worn the device.

**Figure 2:**
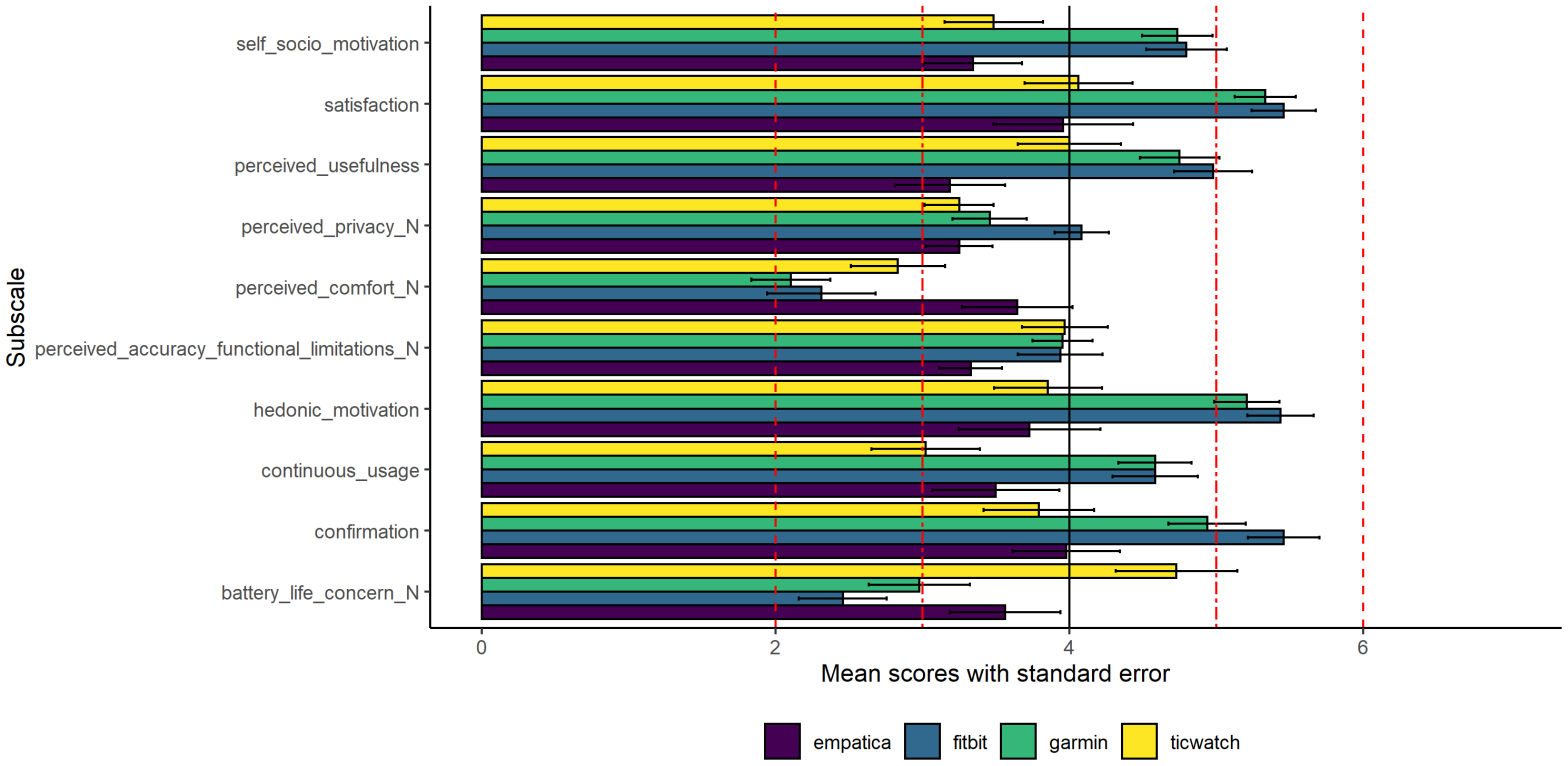
Staff members’ mean scores and standard errors on the subscales of the EECM. *Note that the questions from the subscales denoted with _N were negatively phrased, while the other subscale questions were positively phrased. Similar to the TAM, the EECM also has 10 subscales.

The results for staff members on the EECM are shown in **Figure 2**. For the Fitbit, the mean scores are well above the median for satisfaction, hedonic motivation, and confirmation. However, scores are well below the median for perceived comfort and battery life concern (these two subscales need to be interpreted positively as the questions were phrased negatively). The Garmin has scores well above the median for satisfaction and hedonic motivation, but well below the median for perceived comfort and battery life concern. The Ticwatch has a mean score well below the median for perceived comfort. Lastly, the Empatica did not particularly stand out in terms of continuous use intention.

### 3.4 Patients’ acceptance and continuous use

For patients, we conducted an item-level analysis of both the TAM and EECM (see **Supplementary Materials**). This approach was chosen because it is hard to argue that one or two questions can adequately represent an entire subscale that consists of many items. For both Fitbit and Garmin, over 75% of patients responded positively to several questions: “they liked the idea of using the Garmin and Fitbit”, “found it easy to use”, “thought it was attractive and pleasing”, “thought it was useful for their job”, “felt they could use it anywhere”, “provided them with the desired information and service”, “provided them with a pleasant experience, and it was better than expected”, “found it to be entertaining”, and “thought that the information from the product was correct”. While these percentages were considerably lower for Empatica and Ticwatch, some participants did indicate positive aspects of these devices as well. Over 75% of patients believed that the information from the Empatica was correct, which may be attributed to its purposeful design as a research device, to measure physiological signals as reliably and validly as possible.

## 4 Discussion

### 4.1 Main findings

In this study, a randomized crossover design was employed to assess the usability, acceptance, and continuous use intention of four different wearable devices among both patients and staff members in forensic psychiatric settings. The findings revealed a statistically significant difference in usability between Fitbit and Garmin fitness trackers and two devices that use custom made applications (targeted at gaining insight into physiological reactivity and providing bio-cueing in daily life). Further developments and usability studies are needed to provide users with a similar usability experience as the Garmin and Fitbit fitness trackers. The E4 dashboard and Sense-IT applications were designed to address several challenges in forensic psychiatry, such as emotional self-regulation, (mental) health tracking, behavior modification, providing insight into physiological reactivity, and interoceptive awareness. Achieving comparable levels of usability, acceptance and continuous use intention as commercially available sensors is essential to improve adoption, and research has suggested that gamification and motivation boosting strategies may help to improve uptake and usability (68). In the current study, we aimed to compare multiple devices over extended periods, as earlier research indicated that hands-on experience with wearables was associated with continuous use. Consequently, we limited the scope of the study and did not investigate whether participants appreciated the tailored aspects of the custom made applications for the specific use cases in qualitative research. Rather, we used standardized questionnaires on (continuous) use and acceptance. These aspects are typically well taken care of in commercially oriented wearable technologies.

It is notable that the commercially developed Fitbit and Garmin devices were not above the usability scores that would typically lead people to recommend that technology. Previous research has shown that usability scores should be above ~82 points on the SUS for people to endorse system technology (73). The low scores in usability could seriously hinder the adoption of wearable technology in forensic psychiatry for both staff members and patients, particularly for those patients with mild intellectual disability or borderline intellectual functioning who require user friendly technology. To ensure that participants can derive benefit from the technology (e.g., to gain insight into their physiological reactivity, improve self-regulation or track elements of physical and mental health), it is imperative to develop devices tailored to the unique and personal needs of staff members and patients (12, 13).

Although no formal statistical comparison between staff members and patients was conducted due to violations of several assumptions, SUS-scores indicate a similar trend. Both patients and staff members gave higher scores for Fitbit and Garmin, but lower scores for Empatica and Ticwatch. The difference in SUS scores may be partially explained by the difference in pre-test scores as these were already lower for Empatica and Ticwatch. Although we could not directly compare acceptance and continuous use between patients and staff members due to the use of shortened versions of the questionnaires for patients, the results indicated similar trends for patients as for staff members regarding acceptance and continuous use for all devices.

### 4.2 Strengths and limitations

A particular strength of our current study was the use of a randomized and counterbalanced design that enabled direct comparisons on the usability of the devices, which was a limitation in our previous study (33). Additionally, the shortening of questionnaires for patients with mild intellectual disability or borderline intellectual functioning allowed for easy and time efficient assessment. Staff members found that the administration of these shortened questionnaires were more feasible and comprehensible for patients, thereby reducing the burden of participation and the time needed for questionnaire administration. However, the disadvantage of using these shorter questionnaires for patients was the inability to make direct comparisons for acceptance and continuous use intention between staff members and patients. This limitation stemmed from the fact that patients only received a subset of the questions from the TAM and EECM. Future studies could consider employing the simplified questions for staff members as well to investigate potential differences in acceptance and continuous use intention between the two groups. Although the technology is thought to benefit both staff members and patients, it is important to recognize that the applications and use cases may differ. One aspect for which wearable technology might be useful could be the detection of stress-related problems or sleep problems among staff members. During their admission, patients may display aggression towards staff, leading to potential negative consequences such as symptoms of post-traumatic stress (48, 80, 81). Moreover, recent studies have indicated that aggressive behaviors, including threats, physical aggression, and unwanted sexual approaches, significantly contribute to absenteeism and staff turnover. Additionally, there is substantial evidence linking violence to an increased risk of anxiety, sleep problems, burnout, and depression in staff (80, 82, 83). Wearables could prove especially useful in enhancing the physical and mental health and resilience of staff members.

The four selected devices have different use cases and purposes, which is important to keep in mind when comparing the devices, especially those for which we used (first versions) of custom made software. After consulting with several clinicians and staff members, we believe that these different use cases and devices have potential value for patients and staff members in forensic psychiatric settings. Our current study provides first reference scores on usability, acceptance and continuous use intention. We expect that the potential applications of wearable technology will substantially increase for personalized and tailored use in the coming years (12, 13). The questionnaires on usability, acceptance and continuous use intention provide valuable information on the current state of the wearable technology. The questionnaires clearly indicate what additional work needs to be done before the devices can be implemented on a larger scale.

In a general sense, and as demonstrated by years of research with the SUS, we should aim for technology with usability scores above ~82. Physical activity apps reached a mean usability score of ~83 in a recent meta-analysis (68), and the authors indicated that the popularity of physical activity apps might be due to the gamified nature and motivational features built in these apps. These aspects might also be integrated into the devices and applications used in our study.

A specific limitation of the TAM was enclosed in the Cost subscale, where one question was positively phrased as “CT3: I was able to easily afford this smart watch.”, while the other two were negatively phrased (e.g., CT1: this smart watch was expensive). Calculating a mean score on this subscale effectively influences the interpretation of the question. Given that other questions in the TAM are phrased positively, we would recommend to rephrase all questions in a positive manner, or adopt a similar strategy to the SUS where half of the questions are positively phrased, and half are negatively phrased (37, 74). A comparable scoring system with a maximum score of 100 could also increase comparability on usability, acceptance (and continuous use).

We did not include qualitative questions in the current study as we did in our previous study (33). This decision was made to alleviate the burden on participants who were already asked to wear four devices for of four weeks. Nevertheless, a qualitative evaluation could have provided additional insights into the specific use cases and strengths of the devices that were used. Moreover, we did not include qualitative questions regarding users’ attitudes that are also fundamental aspects related to the adoption of wearable devices (84, 85).

Another limitation is the use of a liberal convenience sampling strategy. We did not include a sample with balanced age, gender, and seniority restrictions, which may have led to selection bias in the current sample. In addition, we did not select specific patient samples, so it is possible that some patients may have been more willing than others to wear the devices. A final limitation is that participants only wore the devices for one week without extensive technology use guidance. For instance, the Sense-IT app was tested in another study where participants were asked to wear the device for longer periods while receiving extensive training on the use of the devices. In those studies, the SUS scores for the Sense-IT app ranged from ~63 to ~76 (65–67). This implies that custom made applications do not readily compare with off the shelve technology (such as Fitbit and Garmin), and needs additional effort for implementation. Extensive guidance and experiential learning might be relevant for special needs populations, such as people with mild intellectual disabilities and borderline intellectual functioning.

### 4.3 Future research

Future research should focus on the validation of the shortened versions of the TAM and EECM in a sample of people with mild intellectual disabilities or borderline intellectual functioning. It is crucial to ensure that the questions are adapted to the needs of those who may have difficulties understanding the questions. Society is becoming increasingly complex, and some people, especially patients with mild intellectual disabilities or borderline intellectual functioning, find it difficult to keep up. We should develop technology that is easy to use, useful, valuable and which has a low cognitive load.

Several researchers have argued that the devices are to be used with caution as the validity and reliability of the algorithms are still questionable (55–57). Also, not all algorithms for artefact detection, stress detection or sleep classification can be validated due to a lack of raw data or proprietary algorithms (42). Open source algorithms and devices such as the custom made applications used in the current study might provide users with information that can be validated. Although the four devices in the current study were carefully selected for use within clinical practice of forensic psychiatric settings, there may still be a degree of subjectivity and selection bias. Future research should prioritize the comparison of additional and multiple devices.

The E4 dashboard and Sense-IT applications were used with custom made software that provides bio cueing (Ticwatch) or combines physiological reactivity information with daily-life situations, events and circumstances (Empatica). The usability scores clearly illustrate that these custom made applications need further improvement. We did not expect that similar usability scores would be obtained as for commercial devices, but future research could explore additional tools to assess and improve usability, perceived ease of use and perceived usefulness, as these constructs are often validated, and indicative of product quality (86). Recent research has suggested that devices preferably have a clear purpose to potentially increase long-term use and user loyalty (87). Both the Empatica E4 dashboard and the Sense-IT app were designed with a very clear purpose in mind, aligning well with earlier recommendations (87).

In the current study, we only used self-report questionnaires and did not consider actual use of the devices. Future studies should consider collecting actual user data, as it might provide additional information that can better align with user needs and preferences (88).

### 4.4 Clinical implications

Wearables provide us with opportunities to understand how patients respond to various (stressful) daily life experiences and (treatment) situations that influence the bodily reactions, physiology and emotional well-being. Together with their therapist, patients can explore whether these technologies provide new insights related to the specific problem the patient is working on. One important implication is the (longer term) usability tailored to each patient’s needs. Some use cases (e.g., tracking specific sleep-stress interactions, evaluating longer treatment effects on self-regulation) might require a different device and measurement duration than others (e.g., physiological reactivity to specific treatment situations, heart rate variability biofeedback). It is important to evaluate device usability beforehand and continuously assess usability to ensure that they remain motivated to work on a specific problem or goal.

The use of wearable technology (e.g., chest straps, ear lobe sensors, wristbands, patches) provides exiting opportunities for delivering continuous (as opposed to episodic) feedback and for interventions on outcomes relevant to the individual. Several systematic reviews showed that wearable technology can have a positive impact on sleep, stress, physical activity, depression, emotional regulation, and cardiovascular and metabolic functioning (14, 20, 24–30). Sleep, stress and physical activity are considered transdiagnostic markers of psychiatric problems (18–20), and can be monitored relatively easy with wearable technology, assuming that the algorithms are accurate and robust (35).

Besides providing information on transdiagnostic markers, wearable technology can offer insights into individual progress or decline (especially throughout treatment, and over treatment sessions), provide just-in-time interventions, or provide insight in daily person-environment interactions and their effects on physiological stress reactivity, cognition and emotion. The challenge is to integrate the wearable technology meaningfully and usefully into clinical practice. Wearable technology has potential for psychological functioning by improving insights, self-awareness, health management, and motivation, but might also have a negative impact on psychological functioning by increasing anxiety, dependency, and worrying (21–23, 42, 66, 89).

Personalized and continuous feedback opens novel opportunities to increase the efficacy of existing treatments and individual functioning (5, 12, 13). However, the current study shows that integration and implementation of this technology is not seamless, and usability, acceptance and continuous use needs improvement. Wearables might provide us with novel opportunities to develop assistive technologies that can be useful for continuous support and just-in-time warnings that can also assist clinicians in providing efficient treatment, reduce physician time, and possibly reducing the cost of healthcare (49, 90).

From a clinical healthcare perspective, Fitbit and Garmin devices are general purpose fitness trackers offering users a range of functions. People can track heart rate (variability), accelerometry, track training progress, recovery, training load, provide reminders, share data with relevant others, and provide composite scores based on physiology that estimate stress, sleep, physical activity, or energy expenditure (91). The uptake of these devices is growing, but there are concerns about their validity, reliability and precision (15, 42, 91). In contrast, the Ticwatch with Sense-IT app (92) has a single purpose function providing real-time bio-cues in the moment that provide insight into changes in heartrate during daily activities. The meaning of the information is not labelled by the device and users can adjust and personalize these settings The Sense-IT provides no interpretation of the changes in the physiology, but lets the user interpret the information. Users can adjust their thresholds for bio-cue information to their liking and add notes regarding their activities. The Sense-IT makes users aware of the bodily changes that occur under different circumstances and under different stressors and events.

On the other hand, E4 dashboard was developed to provide deeper insight into patients’ physiological reactivity throughout the day and synchronizes the information with daily life stressors or events that cause increased arousal. It can be used as a talking board between clinicians and their patients giving them a better understanding of what may cause a bodily reaction of a patient. It may also provide information on under arousal or over arousal of the patient. Further developments of the E4 dashboard include adding open source algorithms for sleep, stress, and physical activity combined with evidence-based information on possible interventions. The current study aimed to establish a reference for different wearable devices and explore applications that might prove useful in forensic psychiatry and other healthcare settings, especially special need samples such as people with mild intellectual disability or borderline intellectual functioning. Different forms and types of wearable technology may prove useful in forensic psychiatry. The wearables used in our study were designed with different goals, applications, and use cases in mind. During treatment, patients have to work on several problems, ranging from trauma to violent behavior, lifestyle coaching, physical and mental health and reintegration. It is unlikely that a single wearable device or application will be useful and valuable for each use case and application. Thus, it is vital to explore different types of wearable technologies to cater the unique needs of patients in forensic psychiatry and other healthcare domains.

## Data availability statement

The raw data supporting the conclusions of this article will be made available by the authors, without undue reservation.

## Ethics statement

The studies involving humans were approved by Ethics (ECSW2020033) and science committee of Radboud University. The studies were conducted in accordance with the local legislation and institutional requirements. The participants provided their written informed consent to participate in this study. Written informed consent was obtained from the individual(s) for the publication of any potentially identifiable images or data included in this article.

## Author contributions

PL: Writing – review & editing, Writing – original draft, Visualization, Software, Resources, Project administration, Methodology, Investigation, Funding acquisition, Formal analysis, Data curation, Conceptualization. MN: Writing – review & editing, Supervision, Methodology, Conceptualization. HN: Writing – review & editing, Resources, Supervision, Methodology, Funding acquisition, Conceptualization. LG: Writing – review & editing, Supervision, Resources, Investigation. SB: Writing – review & editing, Supervision, Resources, Methodology, Conceptualization. RD: Writing – review & editing, Writing – original draft, Supervision, Resources, Methodology, Funding acquisition, Conceptualization.
