# Supplementary material for: Putting the usability of wearable technology in forensic psychiatry to the test: a randomized crossover trial
Source: Front Psychiatry. 2024 Jun 14;15:1330993. doi: 10.3389/fpsyt.2024.1330993 (PMC11212012; doi:10.3389/fpsyt.2024.1330993)
Supplement: Supplementary file 1 [file DataSheet_1.docx]

Supplementary Material

Article Title

P.C. de Looff*, M.L. Noordzij, H.L.I. Nijman, L. Goedhard, S. Bogaerts, R. Didden.

*** Correspondence:** Corresponding Author: [peterdelooff@gmail.com](mailto:peterdelooff@gmail.com)

**Supplementary Figure 1 Proportion and number of responses for staff members on the System Usability Scale questionnaire.**

**
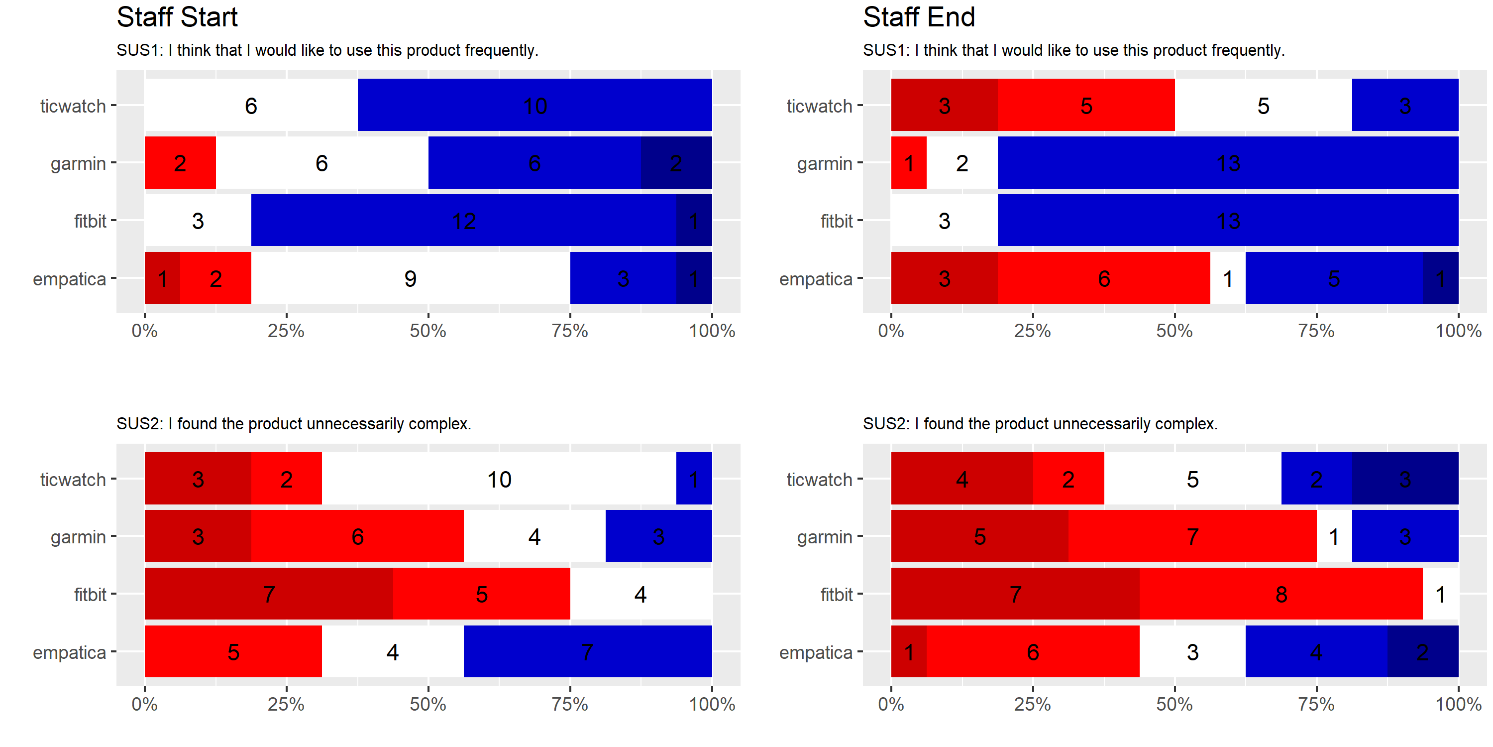

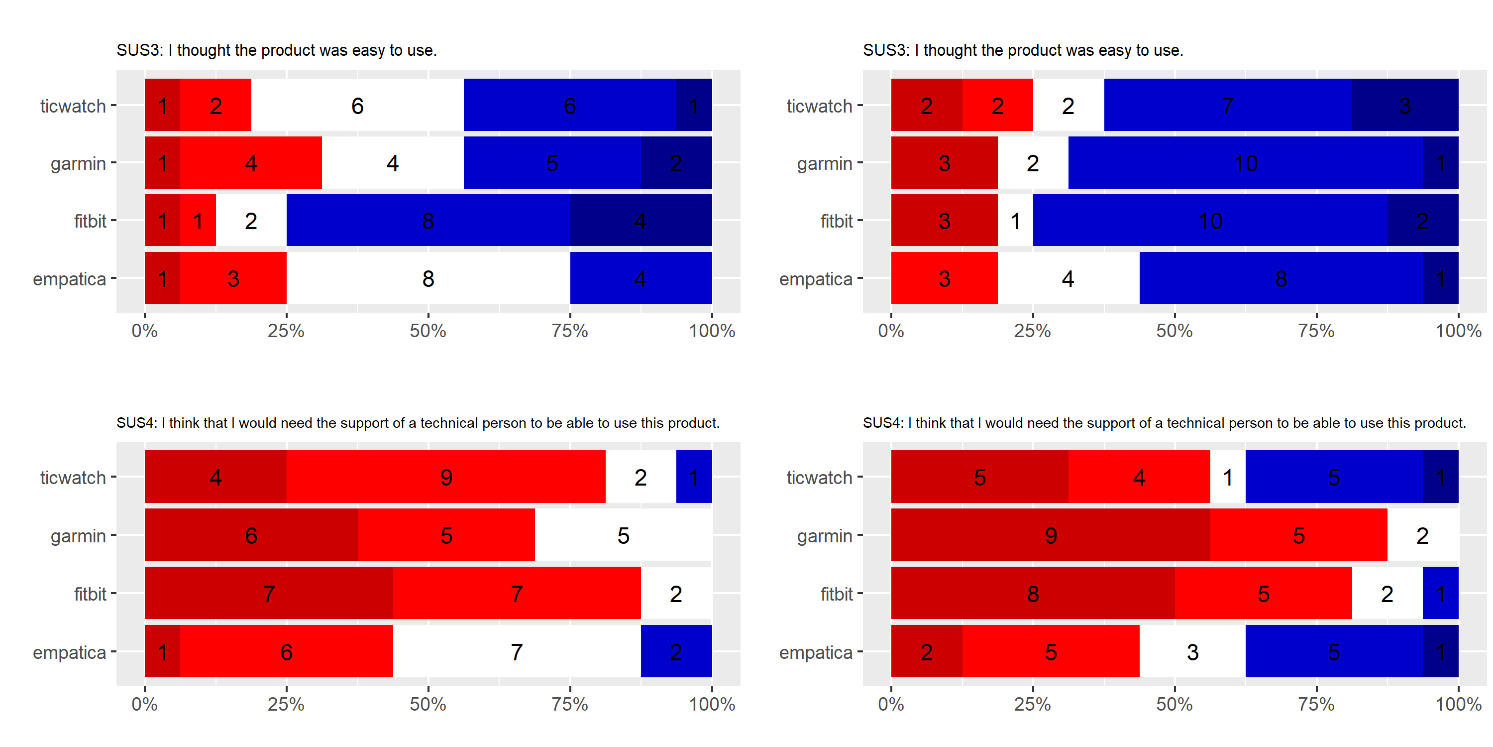

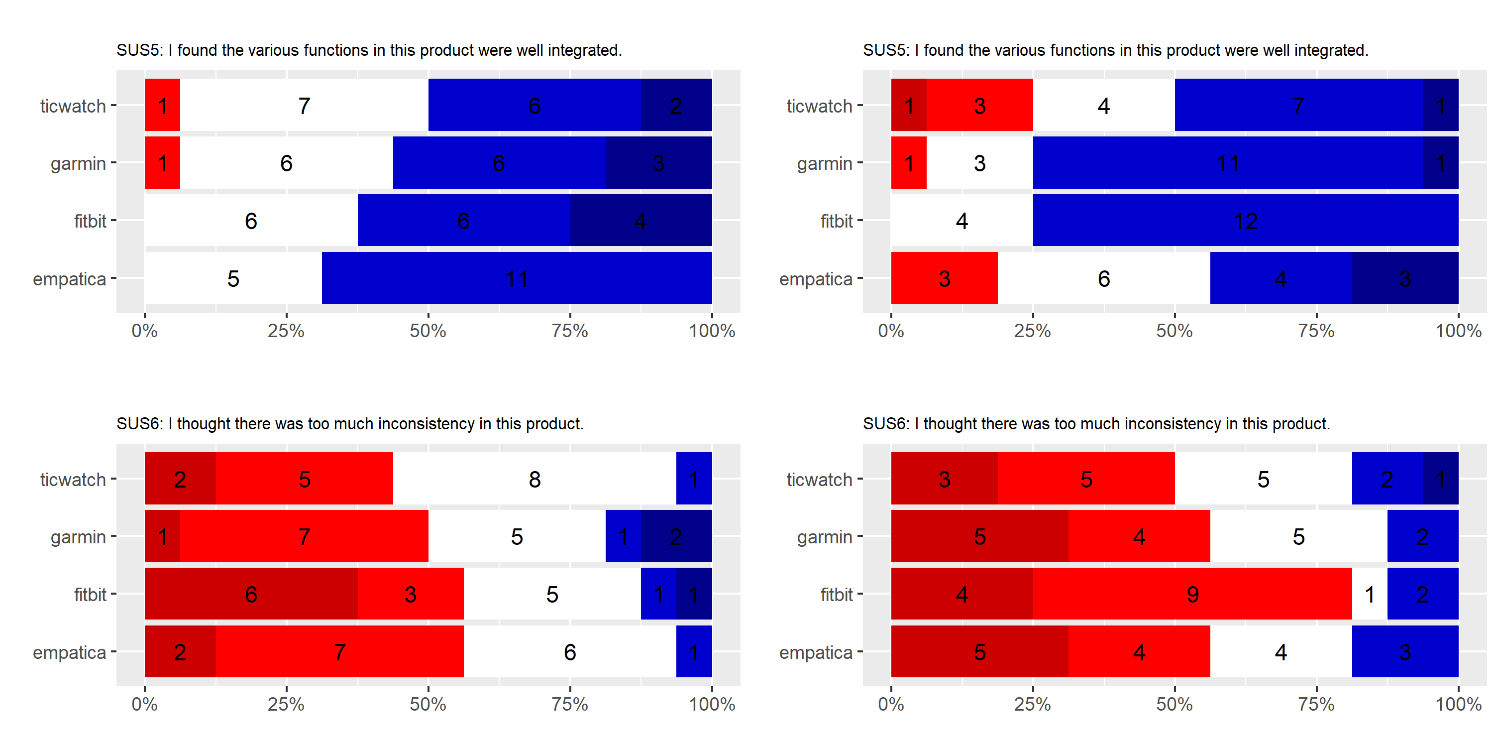

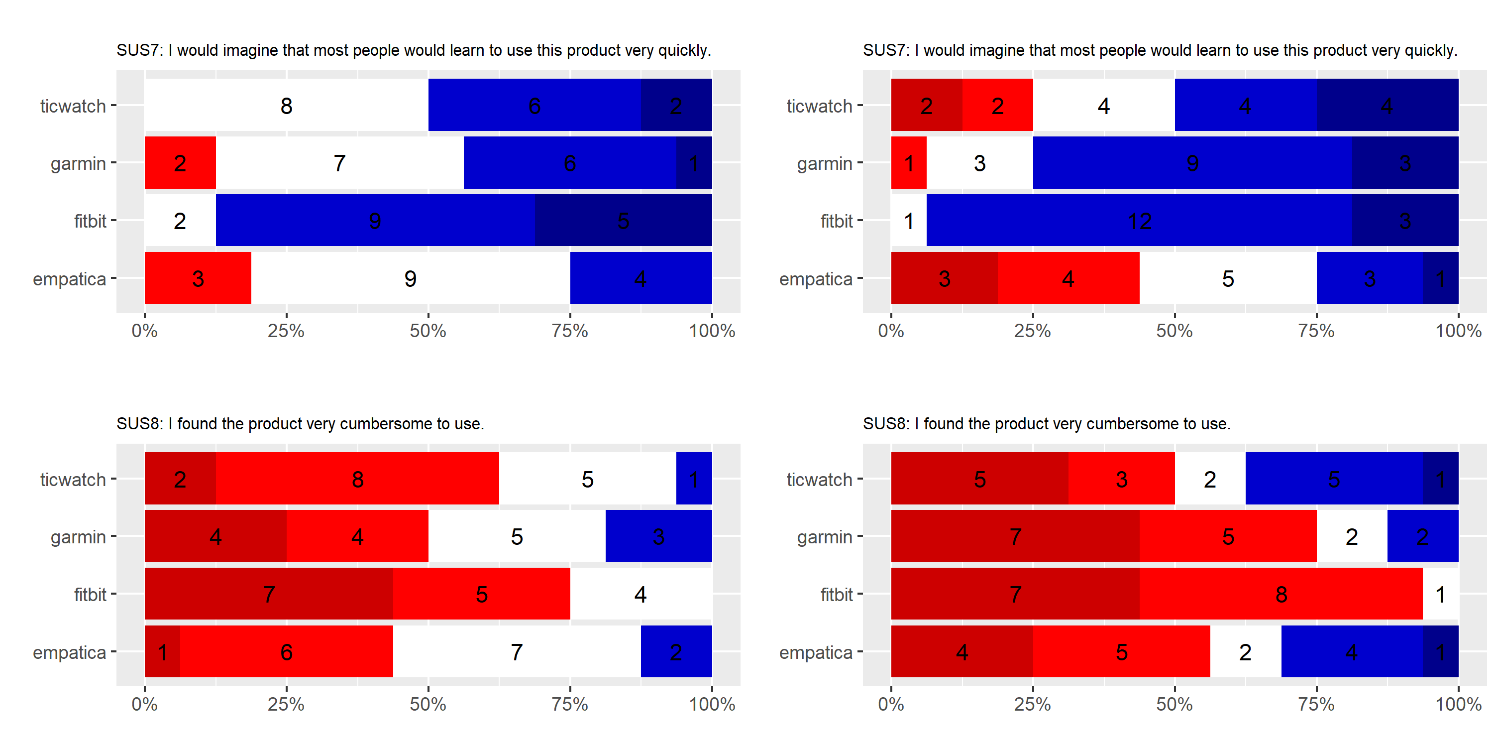

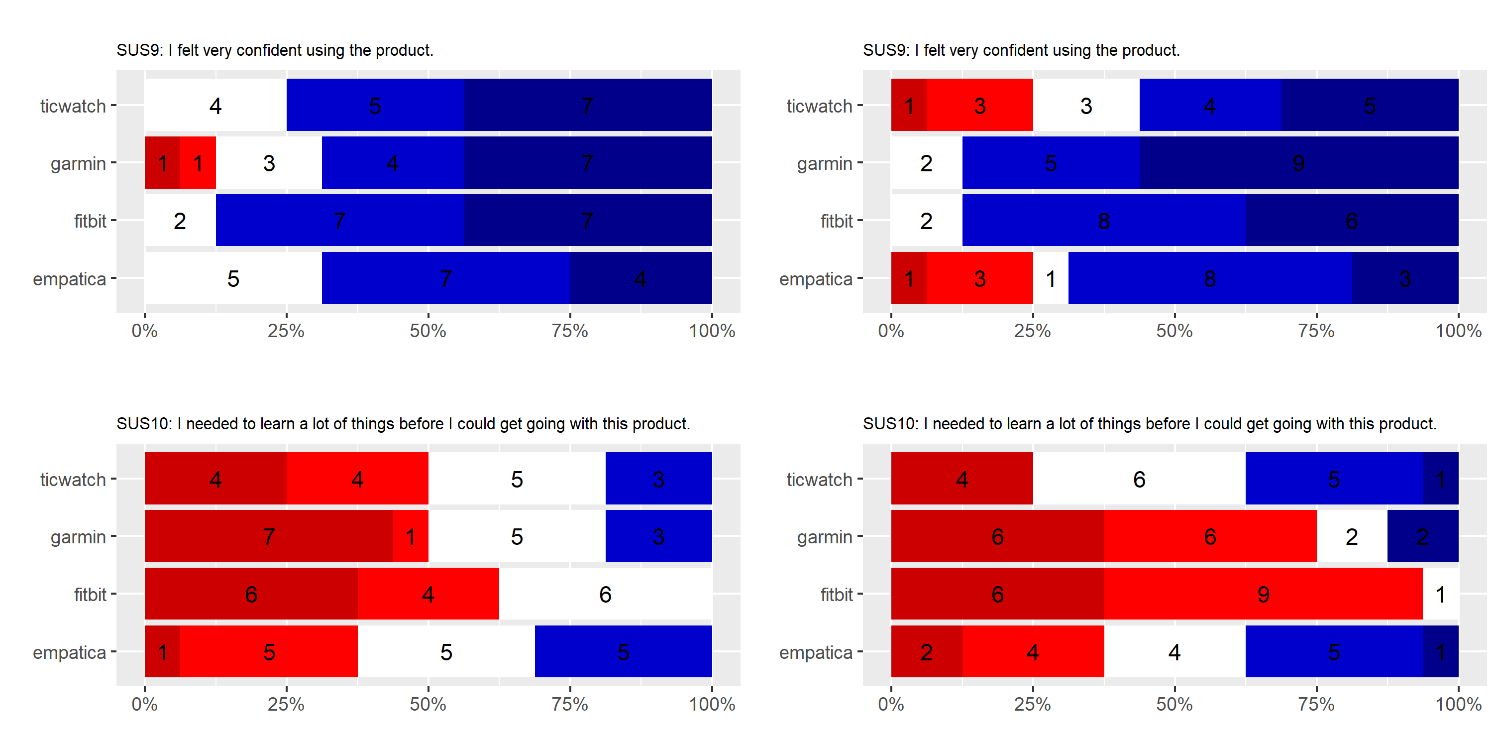
**

**Supplementary Figure 2 Proportion and number of responses for patients on the System Usability Scale questionnaire.**

**
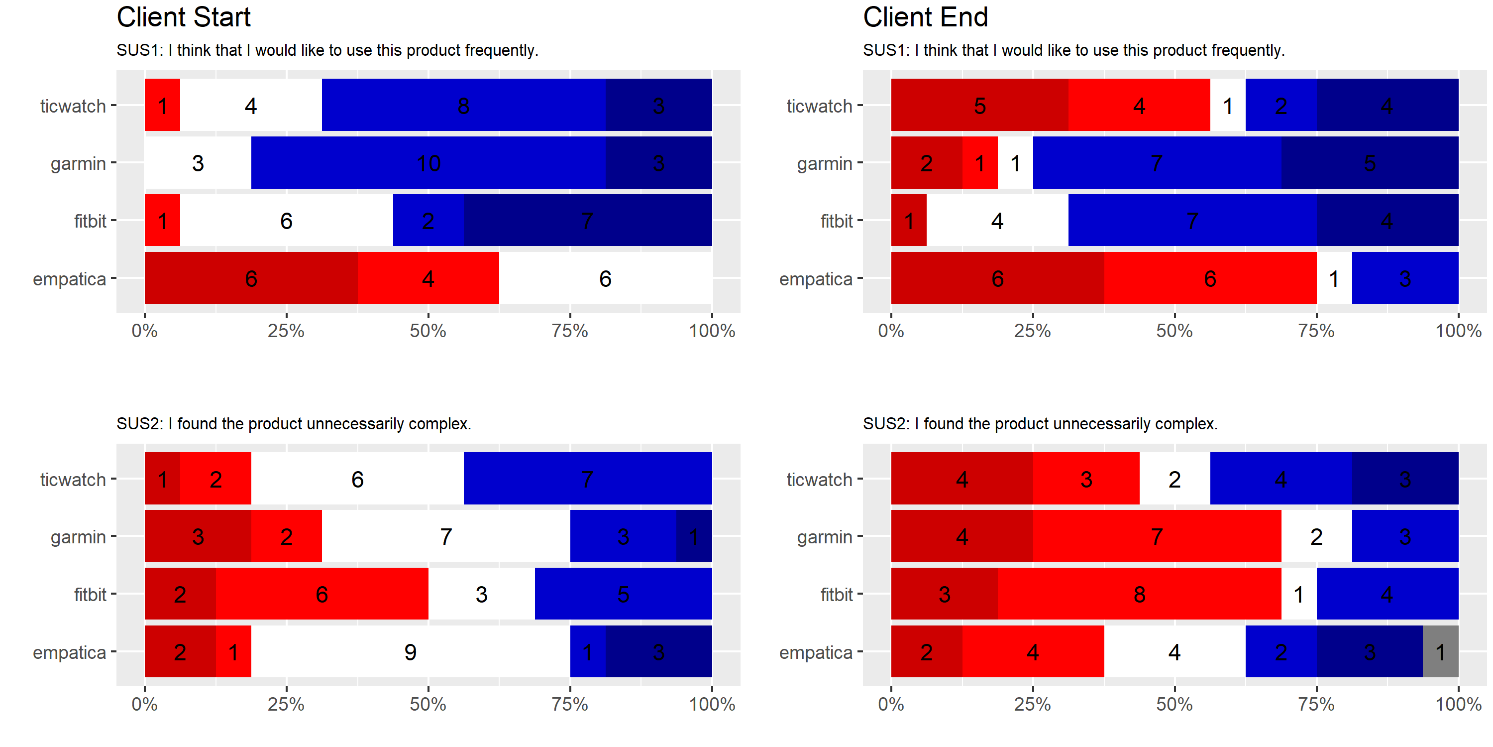

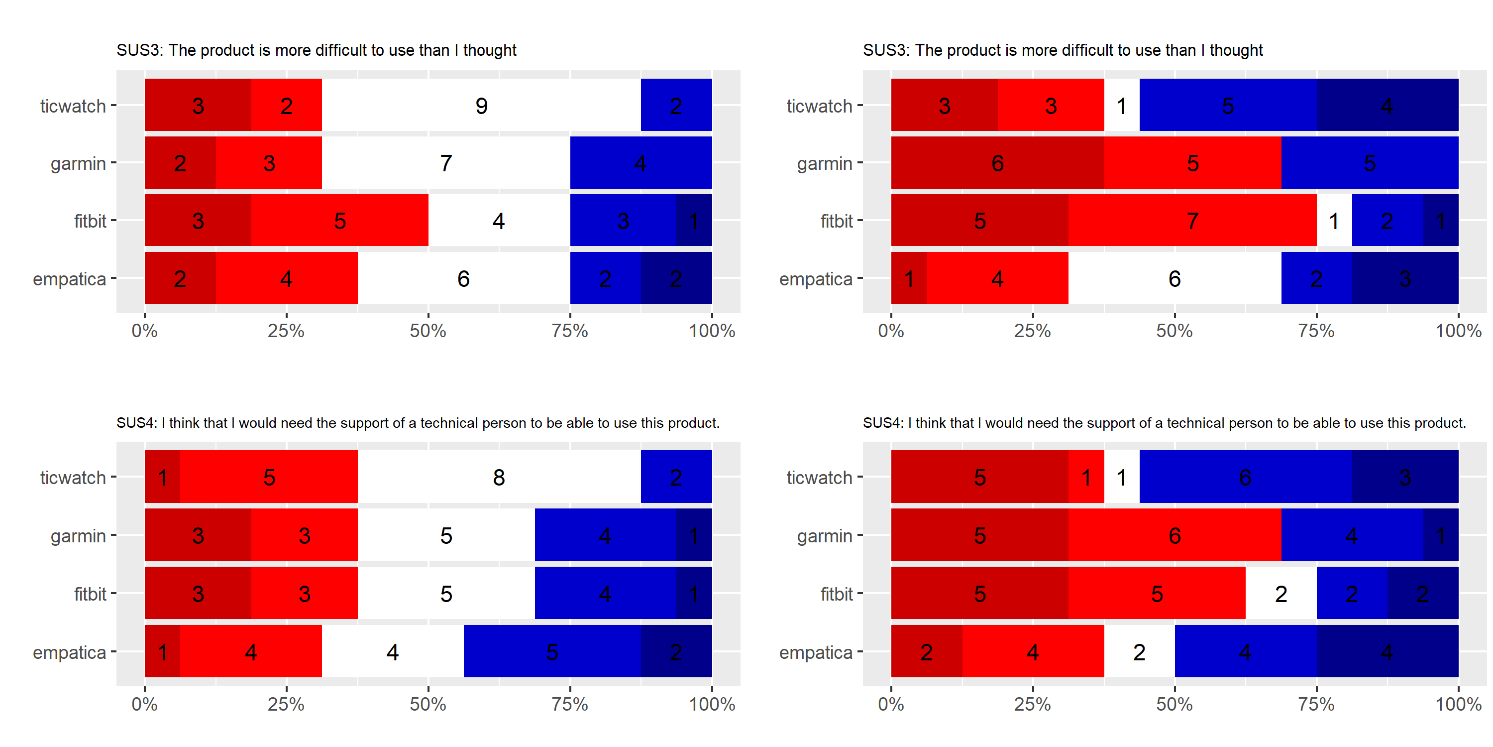

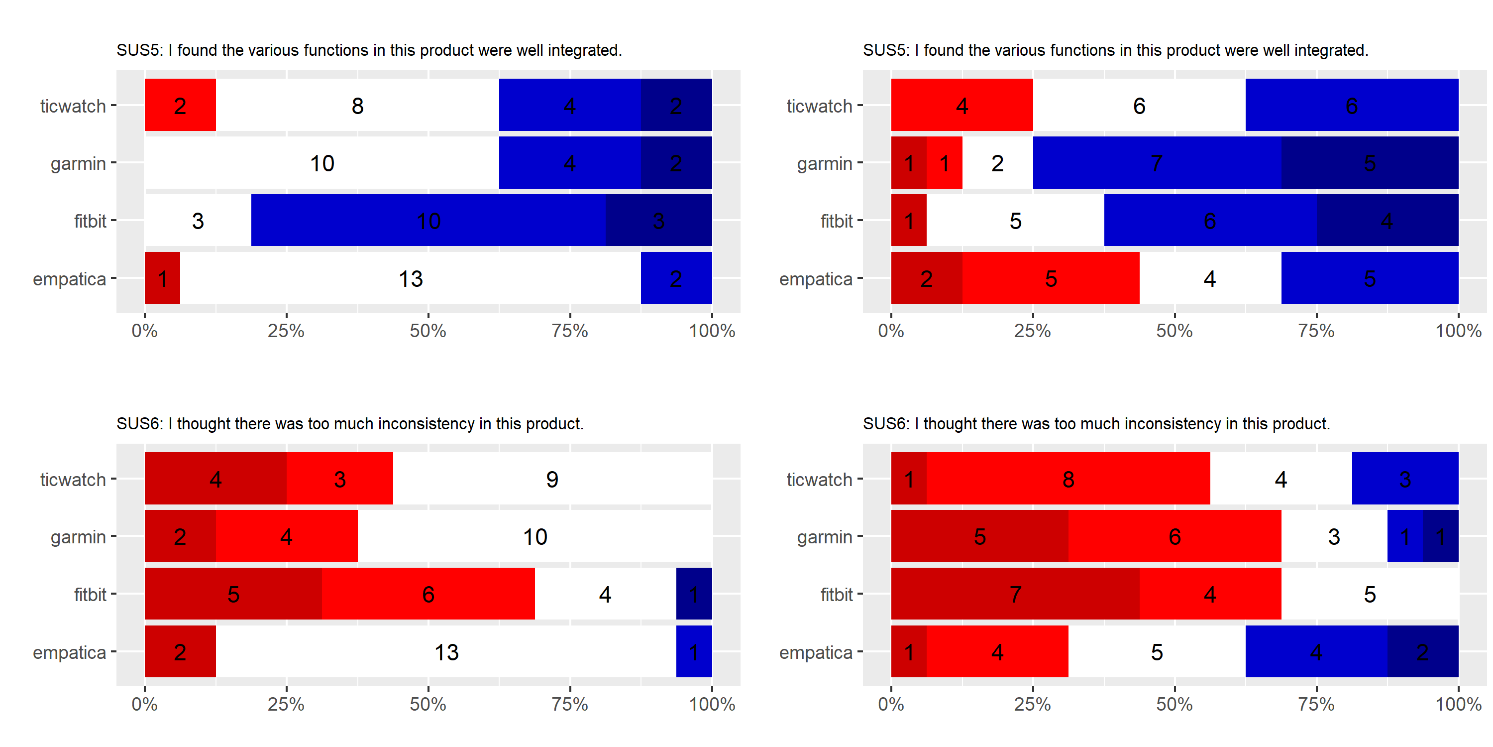

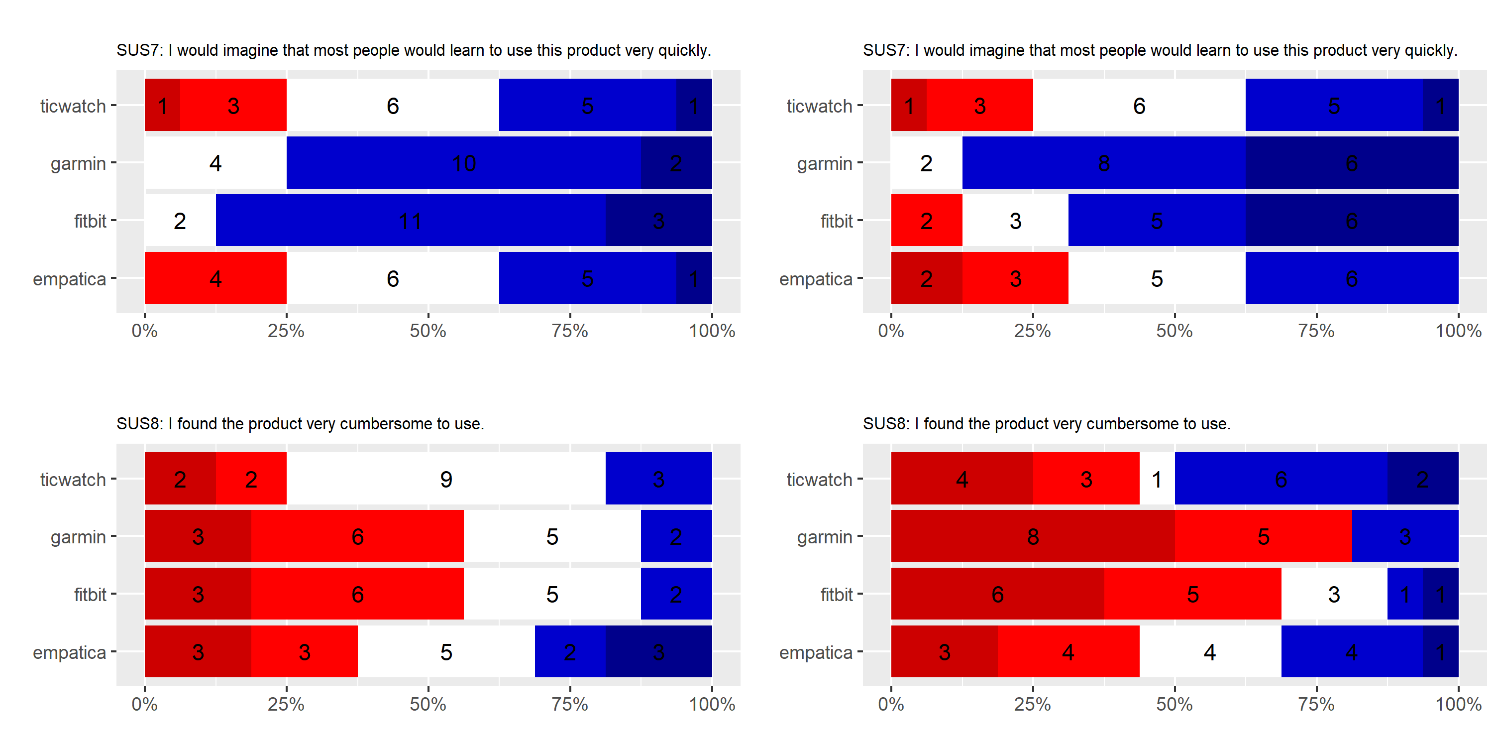

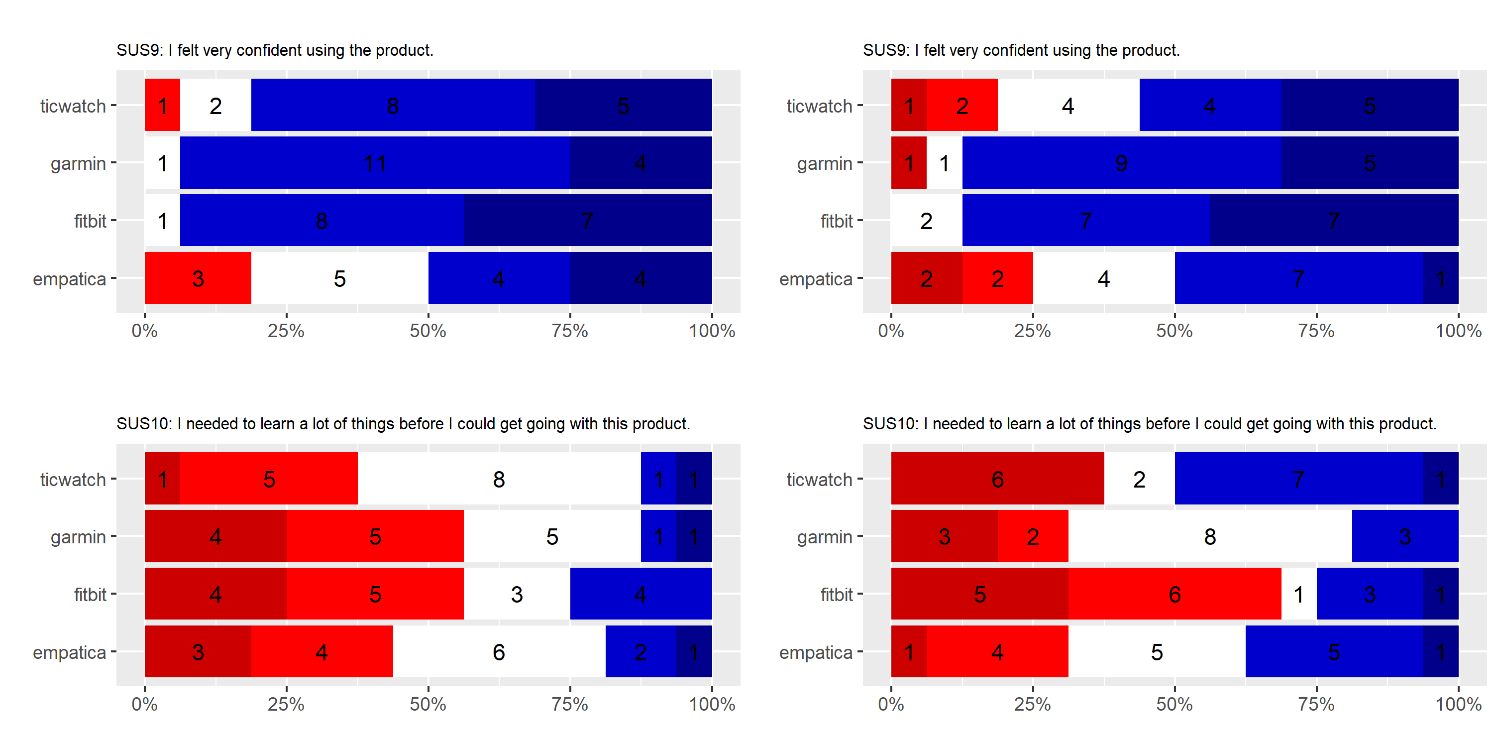
**

**Supplementary Figure 3 Proportion and number of responses for patients on the TAM and EECM.**

**
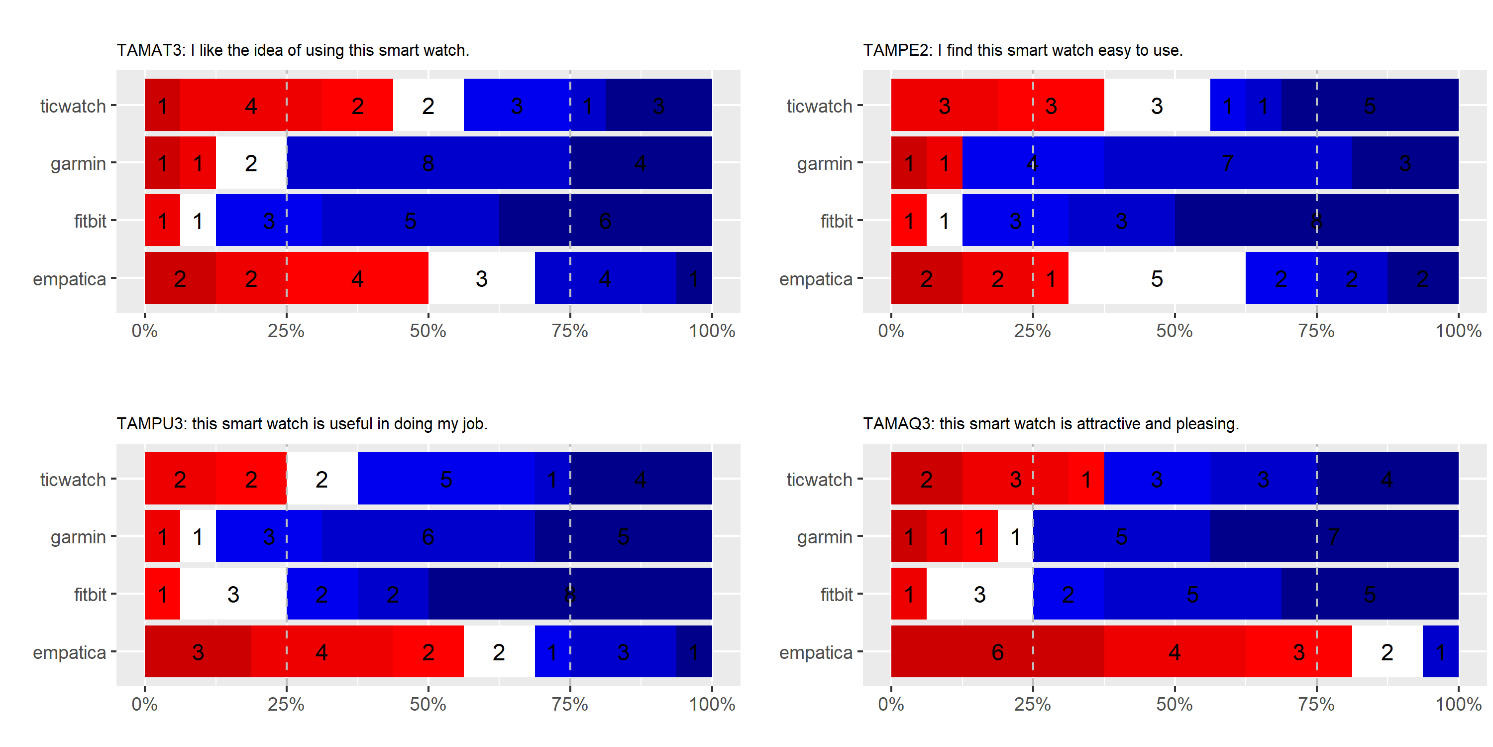

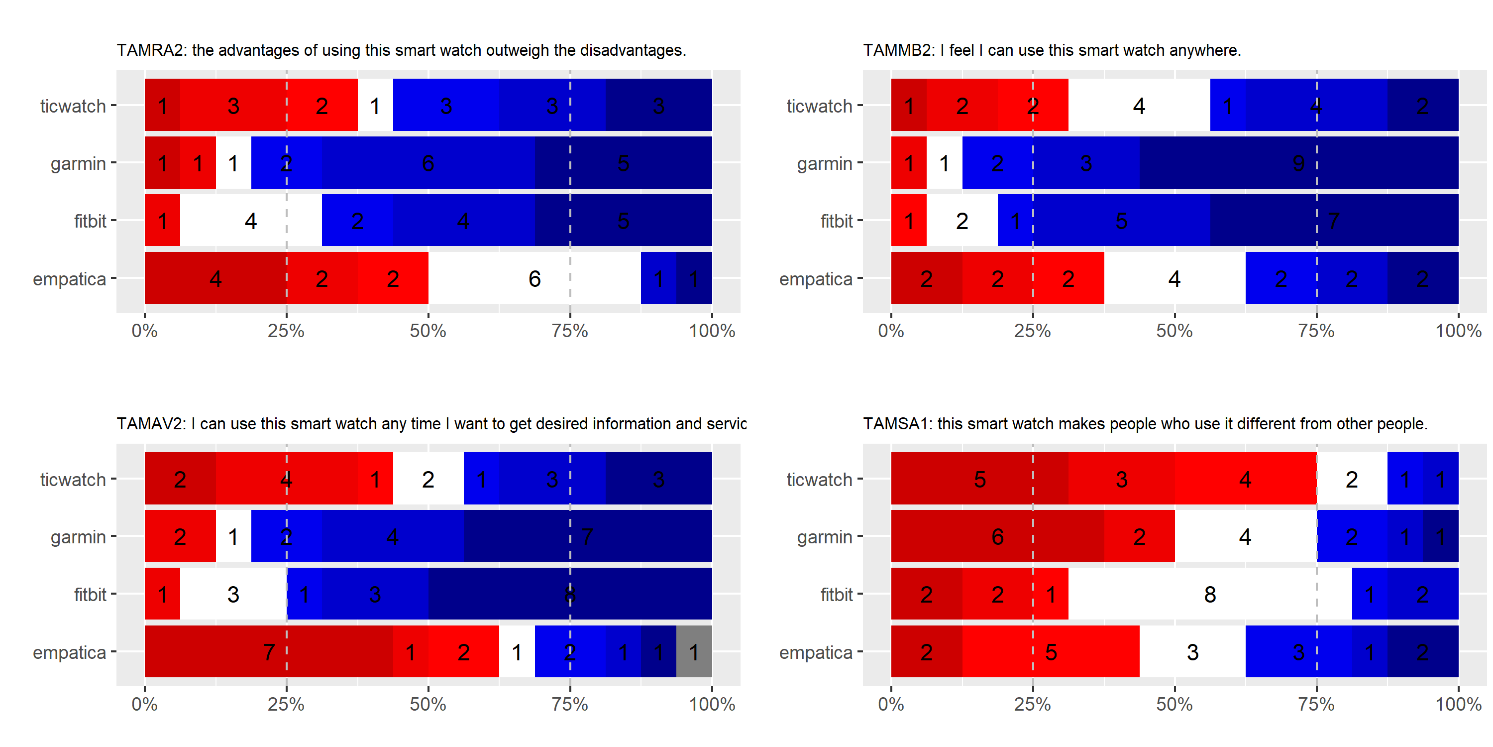

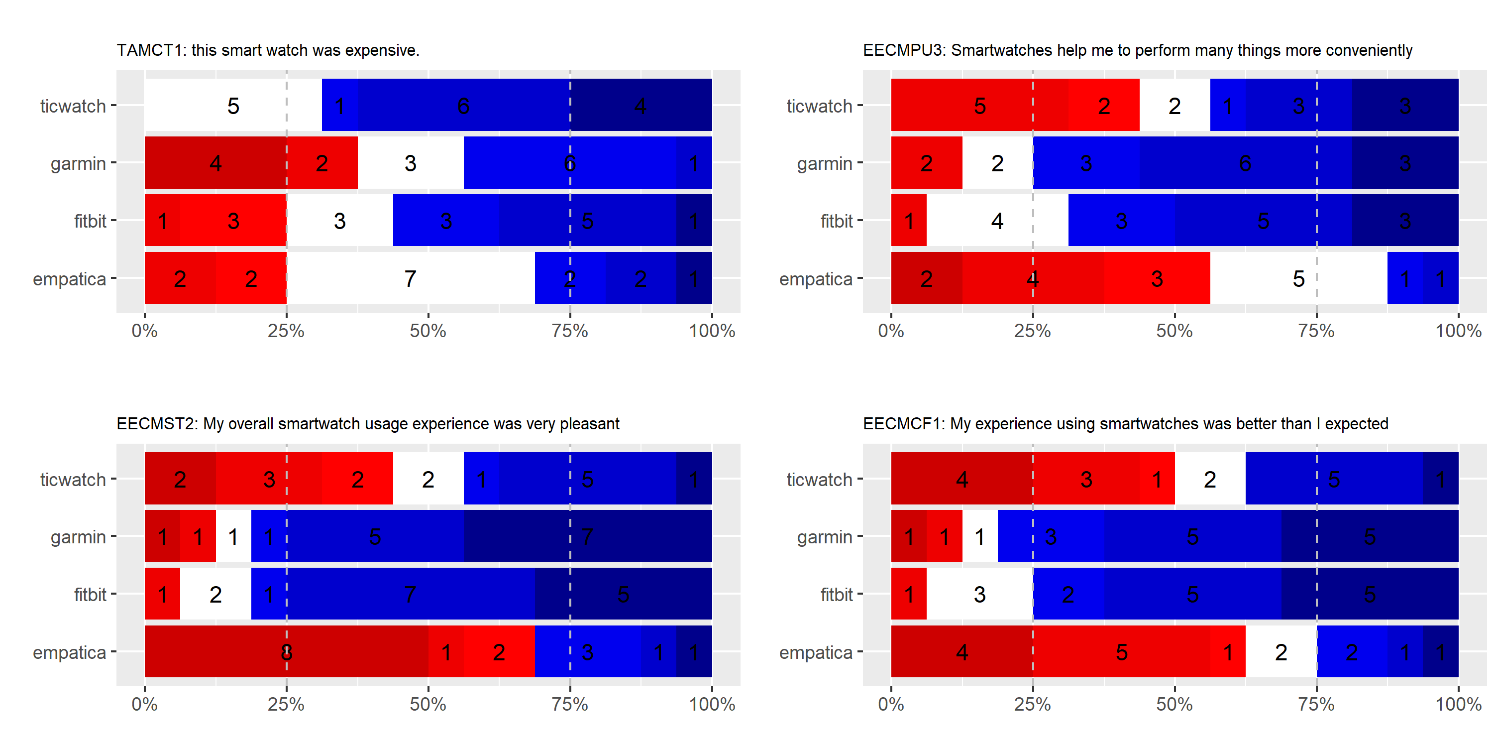

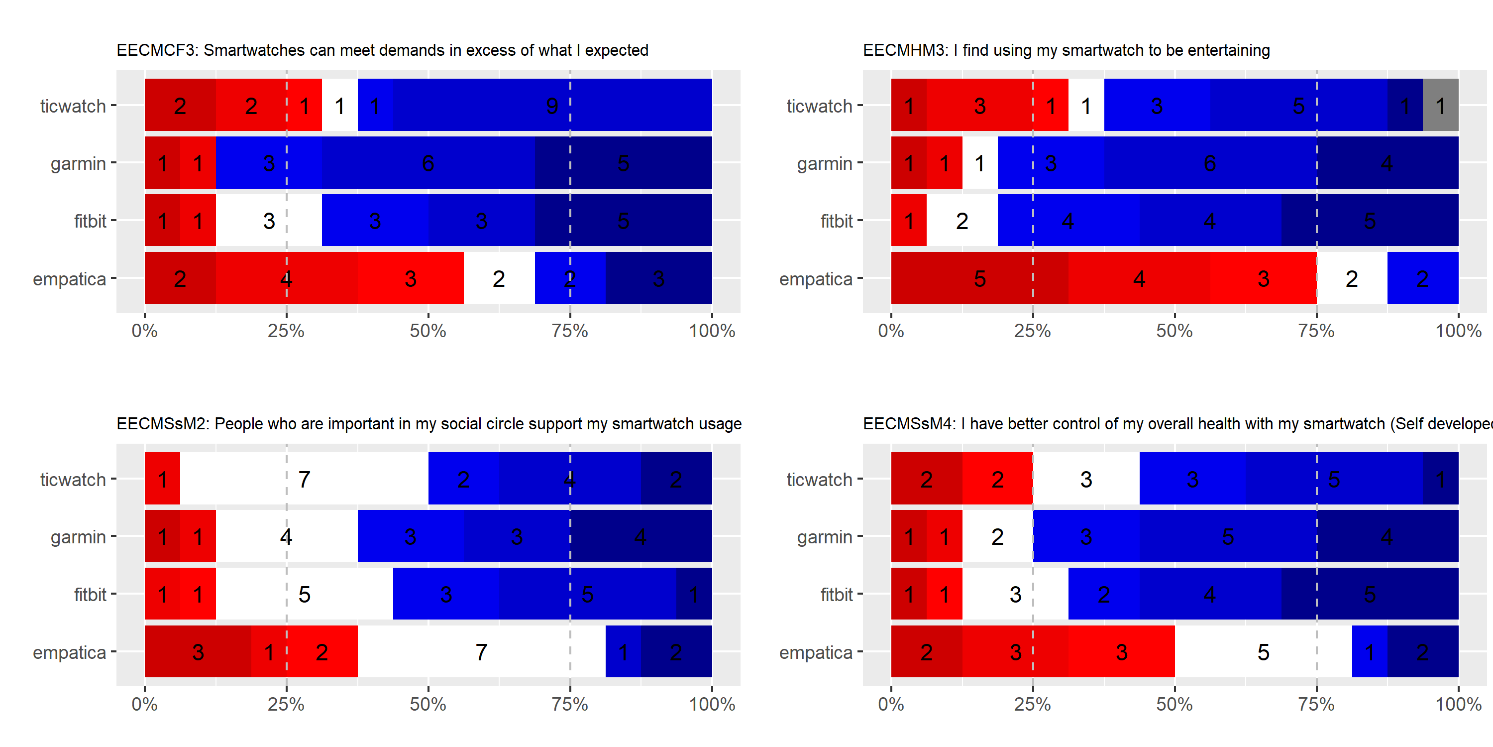

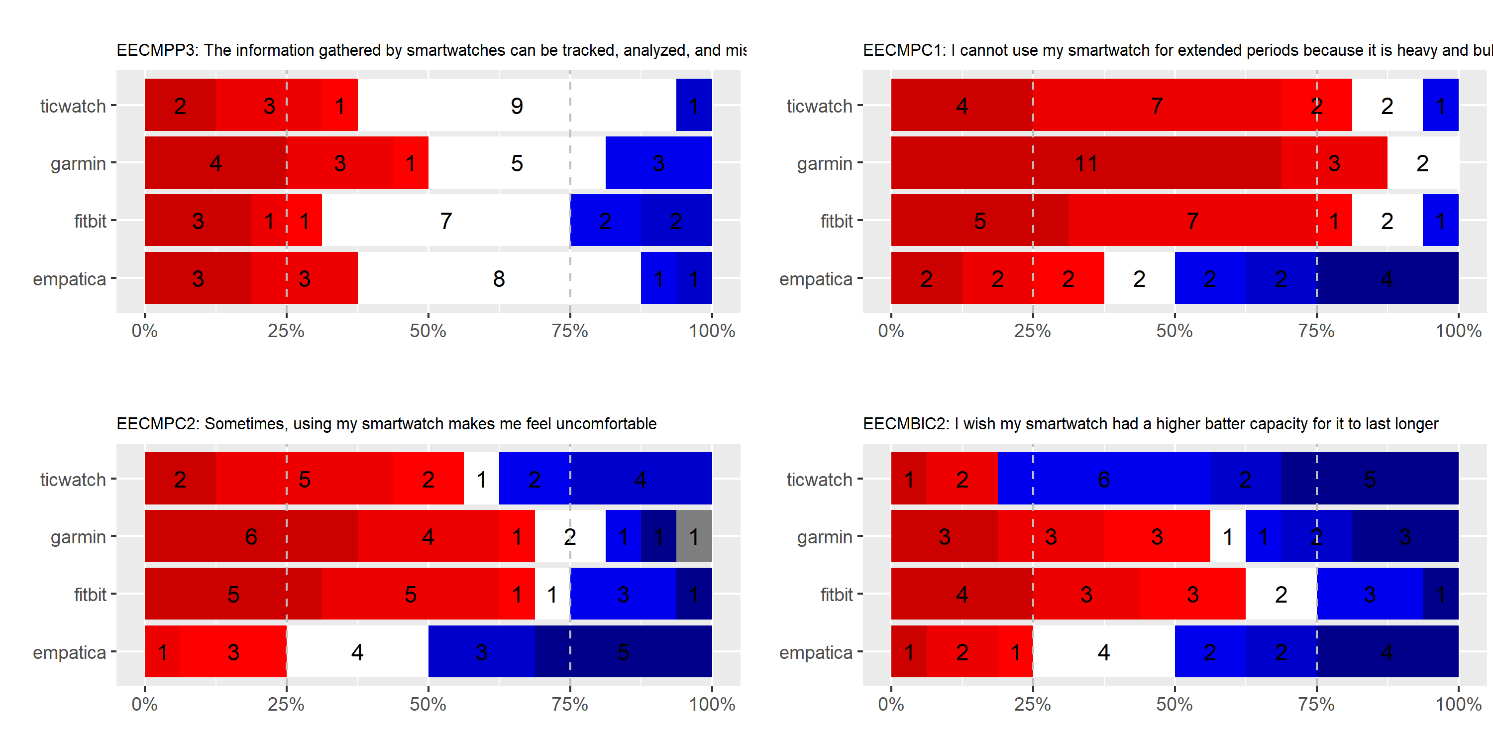

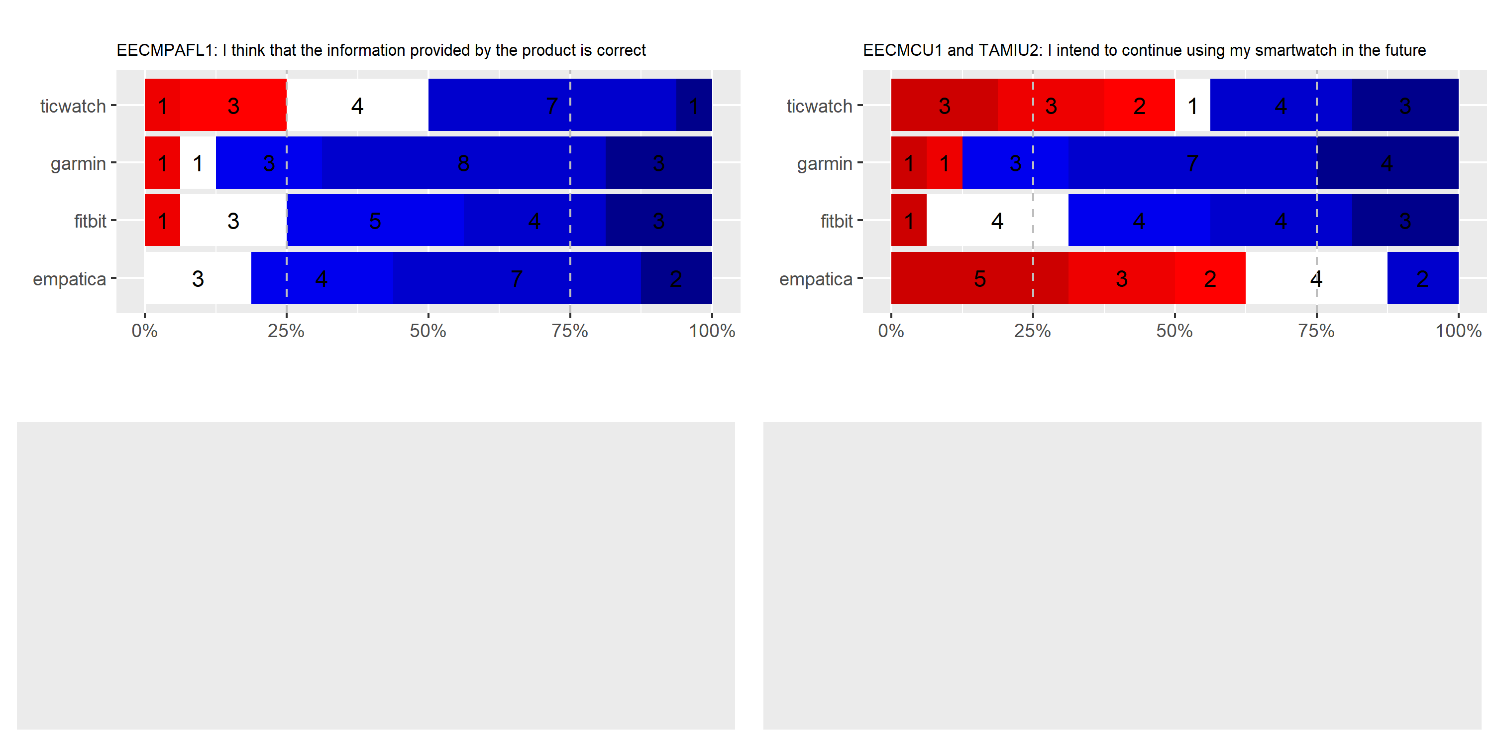
**
